# Deep Learning for Process Monitoring and Defect Detection of Laser-Based Powder Bed Fusion of Polymers

**DOI:** 10.3390/polym18050629

**Published:** 2026-03-03

**Authors:** Mohammadali Vaezi, Victor Klamert, Mugdim Bublin

**Affiliations:** 1 Department of Technical Management, Hochschule Campus Wien, University of Applied Sciences, Favoritenstrasse 226, 1100 Vienna, Austria; mohammadali.vaezi@stud.hcw.ac.at; 2 Department of High Tech Manufacturing, Hochschule Campus Wien, University of Applied Sciences, Favoritenstrasse 226, 1100 Vienna, Austria; victor.klamert@hcw.ac.at; 3 Department of Computer Science and Digital Communications, Hochschule Campus Wien, University of Applied Sciences, Favoritenstrasse 226, 1100 Vienna, Austria

**Keywords:** additive manufacturing (AM), laser-based powder bed fusion of polymers (PBF-LB/P), deep learning, convolutional neural networks (CNN), physics-informed neural networks (PINNs)

## Abstract

Maintaining consistent part quality remains a critical challenge in industrial additive manufacturing, particularly in laser-based powder bed fusion of polymers (PBF-LB/P), where crystallization-driven thermal instabilities, governed by isothermal crystallization within a narrow sintering window, precipitate defects such as curling, warping, and delamination. In contrast to metal-based systems dominated by melt-pool hydrodynamics, polymer PBF-LB/P requires monitoring strategies capable of resolving subtle spatio-temporal thermal deviations under realistic industrial operating conditions. Although machine learning, particularly convolutional neural networks (CNNs), has demonstrated efficacy in defect detection, a structured evaluation of heterogeneous modeling paradigms and their deployment feasibility in polymer PBF-LB/P remains limited. This study presents a systematic cross-paradigm assessment of unsupervised anomaly detection (autoencoders and generative adversarial networks), supervised CNN classifiers (VGG-16, ResNet50, and Xception), hybrid CNN-LSTM architectures, and physics-informed neural networks (PINNs) using 76,450 synchronized thermal and RGB images acquired from a commercial industrial system operating under closed control constraints. CNN-based models enable frame- and sequence-level defect classification, whereas the PINN component complements detection by providing physically consistent thermal-field regression. The results reveal quantifiable trade-offs between detection performance, temporal robustness, physical consistency, and algorithmic complexity. Pre-trained CNNs achieve up to 99.09% frame-level accuracy but impose a substantial computational burden for edge deployment. The PINN model attains an RMSE of approximately 27 K under quasi-isothermal process conditions, supporting trend-level thermal monitoring. A lightweight hybrid CNN achieves 99.7% validation accuracy with 1860 parameters and a CPU-benchmarked forward-pass inference time of 1.6 ms (excluding sensor acquisition latency). Collectively, this study establishes a rigorously benchmarked, scalable, and resource-efficient deep-learning framework tailored to crystallization-dominated polymer PBF-LB/P, providing a technically grounded basis for real-time industrial quality monitoring.

## 1. Introduction

The industrial scalability of additive manufacturing remains constrained by inconsistent part quality, particularly in laser-based powder bed fusion of polymers (PBF-LB/P) [1,2]. Unlike metal powder bed fusion (PBF-LB/M), where defect formation is governed by melt-pool hydrodynamics, PBF-LB/P operates within a quasi-isothermal regime defined by a narrow sintering window between melting onset and crystallization. Within this regime, crystallization kinetics dictate defect evolution. Spatial and temporal variations in thermal history generate shrinkage stresses that manifest as curling, warping, delamination, and coating irregularities [2,3]. These crystallization-driven instabilities fundamentally alter sensing requirements. While visible-light melt-pool imaging is effective in metal systems, it provides limited diagnostic value in PBF-LB/P. Instead, infrared thermography of the evolving thermal field offers a more informative modality for capturing gradual spatio-temporal deviations that precede defect formation [3,4]. Conventional physics-based simulations and post-build inspection remain unsuitable for real-time industrial quality assurance due to computational latency and offline evaluation constraints [5,6]. Deep learning (DL), particularly convolutional neural networks (CNNs), has demonstrated strong defect-detection capability in PBF-LB/M using high-speed imaging [7,8]. However, direct transfer to PBF-LB/P systems is non-trivial. The underlying thermal phenomenology, defect mechanisms, and sensing characteristics differ fundamentally in crystallization-dominated PBF-LB/P [4,9].

**Figure 1:**
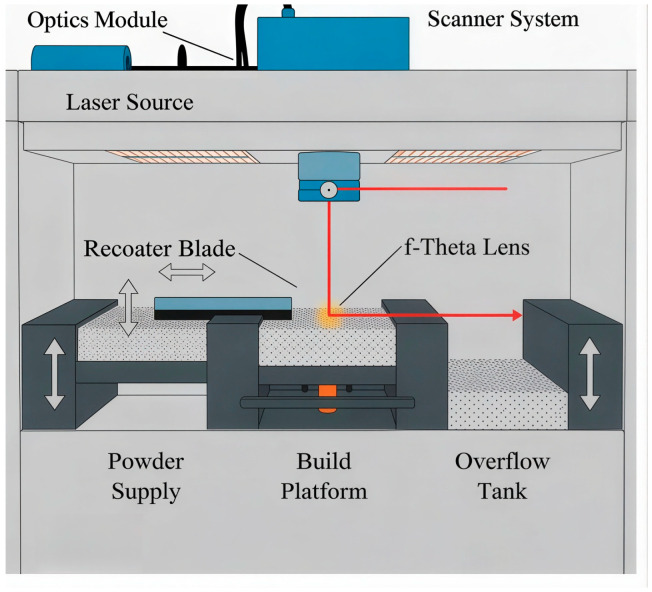
Schematic representation of the laser-based powder bed fusion of polymers (PBF-LB/P) process, including the laser source, optics, and scanner system, f-Theta lens, recoater blade, build platform, powder supply, and overflow tank. Red arrows indicate the laser scanning direction, grey arrows denote recoater movement, and vertical arrows represent the motion of the build platform and powder feed system.

Figure 1 depicts the schematic configuration of a representative PBF-LB/P system, including the laser source, scanning optics, recoating mechanism, build platform, and powder handling units. The process operates under near-isothermal chamber conditions, with the powder bed maintained close to the material’s glass-transition temperature to mitigate thermal gradients and residual stress accumulation.

Beyond thermophysical differences, additional industrial challenges arise. Process stability is influenced not only by crystallization kinetics but also by environmental and material-state variables, including powder aging, humidity fluctuations, cumulative thermal exposure, and inert-gas stability [10]. Publicly available, well-annotated industrial thermal datasets remain scarce, hindering reproducible benchmarking and robust cross-geometry generalization. Furthermore, monitoring solutions must satisfy strict interpretability and computational constraints, enabling sub-2 ms CPU inference on edge hardware integrated into industrial systems operating under closed-loop control [3,11,12].

Although lightweight CNNs and attention-based architectures have reduced inference latency in industrial vision tasks, most are optimized for high-texture natural images or melt-pool-centric metal processes. Thermal-field data in PBF-LB/P exhibit low spatial texture and gradual gradients. Overparameterized architectures designed for high-frequency visual features may therefore introduce representational redundancy or overfitting in this regime, particularly under low signal-to-noise conditions characteristic of industrial thermal imaging. This mismatch underscores the need for domain-aware, resource-efficient modeling strategies.

Unsupervised approaches such as autoencoders (AEs) and generative adversarial networks (GANs) reduce labeling requirements but often suffer from training instability, mode collapse, and limited interpretability in safety-critical environments [13,14,15]. Hybrid CNN-LSTM architectures capture layer-wise temporal dependencies but may become computationally demanding for extended industrial builds comprising millions of frames [16]. Physics-informed neural networks (PINNs) embed governing heat-transfer constraints into the learning objective, improving thermal-field consistency [17,18,19]. While typically requiring substantial offline training, their integration into real-time monitoring frameworks remains an open challenge. Despite increasing interest in data-driven monitoring of additive manufacturing, systematic cross-paradigm benchmarking under realistic industrial PBF-LB/P conditions remains limited. In particular, the interplay between anomaly detection, supervised classification, temporal modeling, and physics-informed regression has not been comprehensively evaluated within a unified experimental framework.

This study addresses that gap. We investigate defect detection in crystallization-dominated PBF-LB/P using synchronized thermal and RGB imaging acquired from a commercial industrial system operating under closed-loop control. We systematically compare unsupervised (AE, GAN), supervised CNN architectures (VGG16 [20], ResNet50 [21], Xception [22]), hybrid CNN-LSTM, and PINN-based approaches to evaluate detection performance, temporal robustness, physical consistency, and deployment feasibility. Particular emphasis is placed on lightweight, domain-aware architectures capable of sub-2 ms CPU inference suitable for industrial edge deployment.

Through this cross-paradigm benchmarking study, the work establishes a rigorously evaluated and practically deployable deep-learning framework tailored to crystallization-driven defect signatures in PBF-LB/P, bridging realistic industrial constraints and scalable in situ quality monitoring.

The principal contributions of this study can be summarized as follows:

- A systematic cross-paradigm benchmarking study of unsupervised, supervised, hybrid, and physics-informed deep-learning approaches under unified industrial PBF-LB/P conditions.
- The development and validation of a lightweight, domain-aware CNN architecture achieving sub-2 ms CPU inference suitable for edge deployment.
- The integration of physics-informed modeling to enhance thermal-field consistency and interpretability.
- Experimental validation on a commercial industrial PBF-LB/P system operating under closed-loop control constraints.

## 2. Methodology

**Figure 2:**
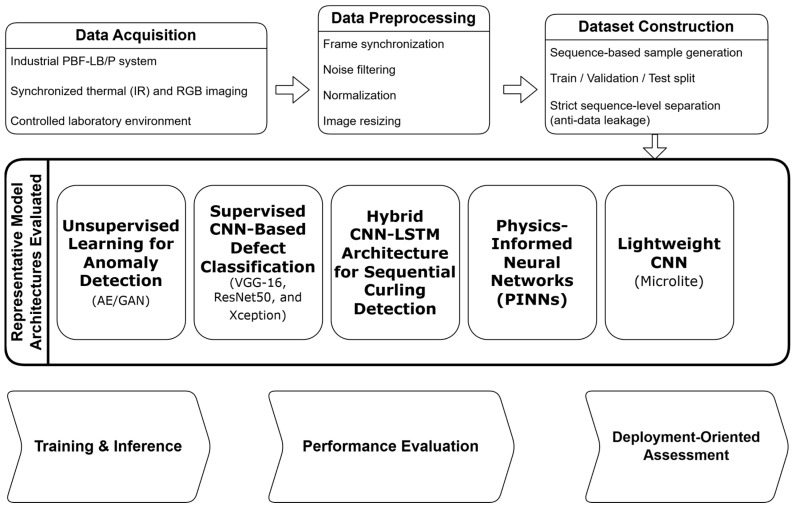
Schematic overview of the experimental workflow, including data acquisition from a commercial PBF-LB/P system operating under closed-loop control, synchronized thermal and RGB preprocessing, build-session–level dataset construction with strict anti-leakage partitioning, and the comparative evaluation of heterogeneous learning paradigms. The framework encompasses unsupervised (AE, GAN), supervised CNN (VGG16, ResNet50, Xception), hybrid CNN-LSTM, physics-informed (PINN), and lightweight domain-aware architectures, with performance assessed in terms of detection accuracy, robustness, and real-time deployment feasibility. Arrows indicate the logical progression of the experimental workflow.

The experimental setup was implemented on an industrial polymer laser-based powder bed fusion platform. An EOS Formiga P110 system, specified in Table 1, was employed for all fabrication experiments. To monitor the spatio-temporal thermal field during printing, an FLIR infrared camera was integrated into the system. An RGB camera was additionally used to acquire visible-spectrum imagery for complementary analysis [23,24]. Figure 2 provides an overview of the complete experimental workflow and model evaluation strategy.

Comprehensive video recordings of the layer-by-layer fabrication process were collected, enabling the extraction of synchronized RGB and thermal image frames. To ensure repeatable, well-controlled experimental conditions, artificial defect scenarios were introduced systematically, including curling (layer warping), localized powder contamination (foreign particle inclusion), and part shifting (geometric misalignment).

All defect scenarios were implemented under authentic industrial operating conditions on a commercial PBF-LB/P system operating under closed-loop control. Importantly, no internal process parameters, exposure strategies, or thermal boundary conditions were modified during the builds. Consequently, the recorded thermal signatures preserve the intrinsic thermo-physical interactions characteristic of industrial PBF-LB/P operation, including the coupled effects of crystallization kinetics and cumulative thermal exposure. While the introduced defects represent controlled experimental scenarios, they reproduce thermally observable failure modes encountered in practice, thereby maintaining industrial relevance while ensuring experimental reproducibility.

### 2.1. Experimental Methods and Data Acquisition

Experimental procedures were performed using an EOS Formiga P110 polymer powder bed fusion (PBF-LB/P) system. A FLIR T420 infrared thermal camera (320 × 240 pixels, 30 Hz) was installed inside the build chamber and interfaced with an external workstation via USB to enable continuous layer-wise thermal acquisition suitable for real-time monitoring [23,24]. The camera continuously monitored the powder-bed surface throughout the fabrication process, generating thermal videos in FLIR (.SEQ) format. These recordings were subsequently converted into individual frame images (.jpg) using ResearchIR software (FLIR Systems, version 4.4). Each frame was timestamped to preserve temporal consistency and stored in three-channel format to ensure compatibility with standard deep-learning pipelines, including convolutional neural networks (CNNs) and long short-term memory networks (LSTMs).

The off-axis FLIR T420 was selected because it reliably captures layer-resolved temperature distributions while maintaining practical integration within a commercial PBF-LB/P system. Compared with RGB or pyrometry-based approaches, infrared thermography provides a balanced trade-off between system complexity and sensitivity to spatio-temporal heat-field dynamics associated with defect formation [25]. In addition to thermal imaging, a compact RGB camera was incorporated into complementary experiments. Independent evaluations on RGB and thermal datasets revealed comparable predictive performance, thereby indicating the robustness of the proposed approach across distinct sensing modalities.

The workflow was implemented using Python 3.10. PyTorch 2.4 and TensorFlow 2.15 were employed according to model-specific requirements. Anomaly detection, supervised CNN classification, and CNN–LSTM architectures were primarily developed in Keras/TensorFlow, whereas physics-informed neural network (PINN) components were implemented in TensorFlow. All experiments were conducted on Ubuntu 21.04 using an Intel Core i7-6700K CPU (4 GHz), 32 GB RAM, and an NVIDIA GTX 1080 GPU (8 GB), reflecting a typical industrial workstation rather than high-performance computing infrastructure.

Comprehensive video recordings of the layer-by-layer fabrication process were collected to extract synchronized thermal and RGB frames. To ensure repeatable, controlled experimental conditions, defect scenarios were introduced systematically, including curling (layer warping), localized powder contamination (foreign particles), and part shifting (geometric misalignment).

All defect scenarios were implemented during standard industrial builds on a commercial PBF-LB/P system operating under closed-loop control. No internal exposure parameters, scanning strategies, or thermal boundary conditions were modified. Consequently, the recorded thermal signatures preserve intrinsic multi-factor interactions among crystallization kinetics, environmental stability, and cumulative thermal history characteristic of real industrial operation. While these defects represent controlled scenarios, they reproduce thermally observable failure modes encountered in practice, thereby balancing experimental reproducibility with industrial relevance.

To ensure reproducibility, random seeds were fixed across all experiments. The overall framework was structured into sequential experimental phases, progressing from unsupervised anomaly detection to supervised classification, temporal modeling, and physics-informed hybrid approaches. This staged design enabled systematic comparison of detection accuracy, interpretability, and computational efficiency across modeling paradigms.

### 2.2. Data Preprocessing and Anti-Leakage Protocol

A strict anti-leakage protocol was implemented prior to model training to ensure unbiased evaluation and scientific rigor. Particular attention was given to preserving temporal integrity and preventing information flow between dataset subsets. Preprocessing steps included sequence-level partitioning, normalization based solely on training data statistics, and controlled augmentation strategies applied exclusively to training samples. The final dataset comprised approximately 76,450 synchronized frame samples acquired under industrial PBF-LB/P conditions. The class distribution reflects a realistic production imbalance, with defective events constituting a minority class. To address this, weighted cross-entropy loss and class-aware sampling strategies were applied during training.

#### 2.2.1. Data Partitioning and Sequence Isolation

All captured thermal and RGB frames were organized into temporally coherent build-session sequences, each treated as an indivisible unit to prevent temporal fragmentation across partitions. Accordingly, dataset partitioning was performed at the build-session level to preclude temporal dependencies between subsets. An 80/20 split was defined a priori, with the 20% portion reserved as an independent test dataset and strictly isolated from all model development stages, including hyperparameter optimization and threshold calibration. The splits were generated deterministically using a fixed random seed to ensure reproducibility. Integrity verification confirmed the absence of frame-level and sequence-level overlap between subsets, thereby mitigating the risk of data leakage.

#### 2.2.2. Normalization and Statistical Independence

Normalization parameters, specifically the mean and standard deviation per RGB channel, were computed exclusively from the training subset defined by the predetermined data split. These statistics were subsequently applied to the validation and test datasets without recalculation. This procedure preserved statistical independence and prevented normalization-induced leakage across partitions.

Data augmentation techniques, including horizontal flipping, ±10° rotation, and minor color jitter, were applied solely to training samples. Validation and test data remained unaltered to ensure unbiased performance evaluation.

#### 2.2.3. Verification and Audit Compliance

An internal verification procedure was conducted to confirm compliance with the predefined anti-leakage safeguards:

- Complete separation of build-session sequences between training, validation, and test subsets;
- Normalization statistics fitted exclusively on the training data;
- Data augmentation restricted to the training subset;
- Strict isolation of the independent test dataset from all model development stages;
- Validation data used solely for hyperparameter optimization;
- Deterministic split generation using fixed random seeds;
- Full traceability of partitioning and preprocessing parameters.

All criteria were satisfied, confirming zero overlap, full statistical isolation, and complete test-set integrity. Observed model performance therefore reflects genuine generalization to unseen data rather than latent data redundancy.

### 2.3. Phase I: Experimental Framework for Unsupervised Anomaly Detection

The initial experimental phase utilized an unsupervised learning framework to evaluate early anomaly detection in thermal images from polymer powder bed fusion (PBF-LB/P) processes. This phase compared two neural network architectures, the autoencoder (AE) [26] and the Generative Adversarial Network (GAN) [13], alongside a baseline K-Means clustering approach [27,28]. Autoencoders were selected as a baseline unsupervised method due to their ability to learn compact latent representations of normal thermal patterns and detect deviations without requiring labeled defect data. K-Means clustering, as an unsupervised learning method, can help reduce the need for labeled data and can be combined with supervised learning in a semi-supervised learning approach. GANs can be used to learn latent representations and to augment data, thereby increasing the number of training samples.

The autoencoder implemented a symmetric convolutional encoder–decoder network and was optimized using Mean Squared Error (MSE) loss to reconstruct normal thermal frames. The GAN architecture consisted of a generator and a discriminator trained competitively to synthesize realistic, defect-free thermal images.

For the K-Means algorithm, feature vectors were reduced using principal component analysis (PCA) to serve as a comparative unsupervised baseline [28]. Performance metrics included reconstruction error (MSE) as an anomaly indicator, accuracy on a small labeled subset, and the Silhouette Coefficient (s) to assess clustering quality.

Additionally, contour-based feature extraction was evaluated for its capacity to identify curling regions independently of object geometry. This approach employed the Canny edge detection algorithm from the OpenCV library, applying dynamic maximum and minimum thresholds to delineate object boundaries and adjacent powder regions.

### 2.4. Phase II: Supervised CNN Training Procedure

Supervised learning, in general, provides the best performance but needs (a lot of) annotated data. Supervised thermal image classification was employed in the experimental setup detailed in Section 2, building upon the framework developed during Phase I. Domain experts manually annotated each frame as either “OK” (nominal layer) or “DEF” (curling or delamination). Frames labeled DEF correspond to defective layers characterized by temperature distortions and surface warping, whereas OK frames represent uniform, defect-free layers. This annotation protocol enabled supervised convolutional neural networks (CNNs) to learn discriminative thermal patterns associated with curling formation during the PBF-LB/P process.

The experimental workflow comprised three primary steps:

(1) Baseline training and evaluation of VGG-16, ResNet50, and Xception convolutional neural networks (CNNs) [20,21,22,24]
(2) Validation of predictions on both individual and batch samples; and
(3) Generation of Gradient-weighted Class Activation Mapping (Grad-CAM) visualizations to enhance interpretability [29].

Training followed the configuration described in Section 2.1, employing binary cross-entropy loss with the Adam optimizer [30], batch size of 32, and early stopping. Class imbalance was addressed using weighted loss functions and controlled augmentation applied exclusively to the training subset.

VGG-16, ResNet50, and Xception were included due to their strong performance and the availability of pretrained models, which enable transfer learning. They have also been used for defect detection in PBF-LB/P [31].

### 2.5. Phase III: Sequential Modeling Using a Hybrid CNN–LSTM Architecture

In the third phase, a hybrid CNN-LSTM architecture was developed to extend the static VGG-16 classifier, enabling temporal sequence recognition [20,32].

While Phase II addressed frame-level classification, this phase explicitly modeled the progressive evolution of curling across consecutive layers, reflecting its multi-layer development mechanism in PBF-LB/P processes. The VGG-16 backbone, with its fully connected head removed, was used as a feature extractor within a Time-distributed wrapper. Extracted features were subsequently passed through a Flatten layer and a 256-unit LSTM layer, followed by fully connected layers with a final softmax activation.

This architecture enabled the model to process short sequences (10 × 224 × 224 × 3) instead of individual images. The sequence length was selected as a compromise between capturing short-term temporal evolution and maintaining computational feasibility. Sequences were labeled as DEF (curling) or OK (normal) based on layer-level annotations. Training and evaluation followed the experimental protocol described in Section 2. Class imbalance was addressed using weighted loss functions and controlled sampling applied exclusively to the training subset to preserve statistical independence [33]. This configuration prioritized stable convergence while limiting false-positive detections in an industrial monitoring context.

**Figure 3:**
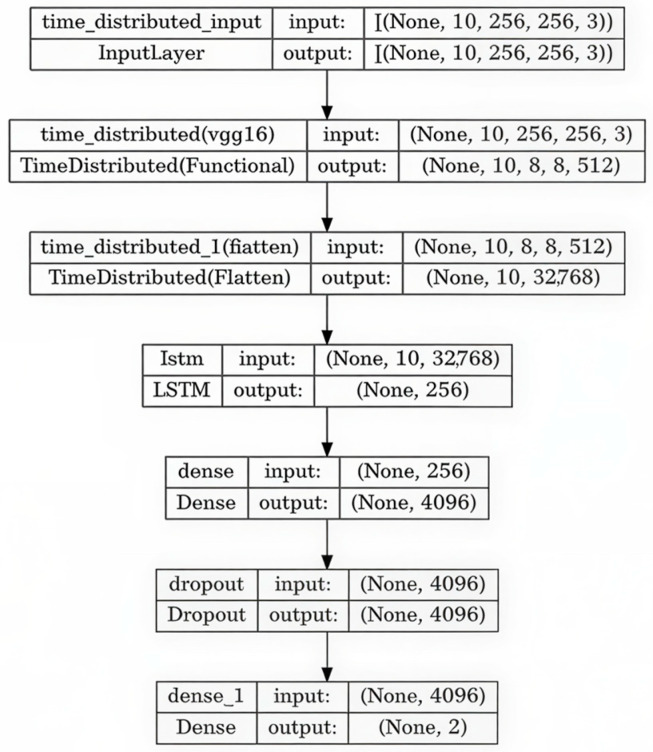
CNN LSTM hybrid model. Arrows indicate the data flow between layers.

Regularization techniques, including Dropout (0.5), early stopping, and controlled augmentation, were implemented to mitigate overfitting. The model was implemented using Keras/TensorFlow and trained on the same industrial workstation configuration described previously. Each experiment was conducted for up to 90 epochs with a batch size of 32 and categorical cross-entropy loss. Sequence-level labels were used, and predictions were generated at the sequence level rather than per individual frame. The proposed CNN-LSTM architecture is illustrated in Figure 3.

### 2.6. Phase IV: Application of Physics-Informed Neural Network (PINN) for Predicting Thermal Fields

During the fourth phase, a Physics-Informed Neural Network (PINN) was implemented to integrate heat transfer equations with deep-learning techniques. This methodology facilitated spatio-temporal temperature prediction in the polymer laser powder bed fusion (PBF-LB/P) process [17]. The PINN is employed for physics-consistent thermal field prediction and interpretability rather than for closed-loop real-time process control. The framework consisted of three primary components: a physics-based heat conduction model, a deep neural network for data-driven learning, and a physics-informed loss function that combined the two domains.

The governing heat transfer equation is as follows: [5]

(1) ρcₚ (∂T/∂t)=∇·(k∇T)+Q(x,y,z,t)

**Figure 4:**
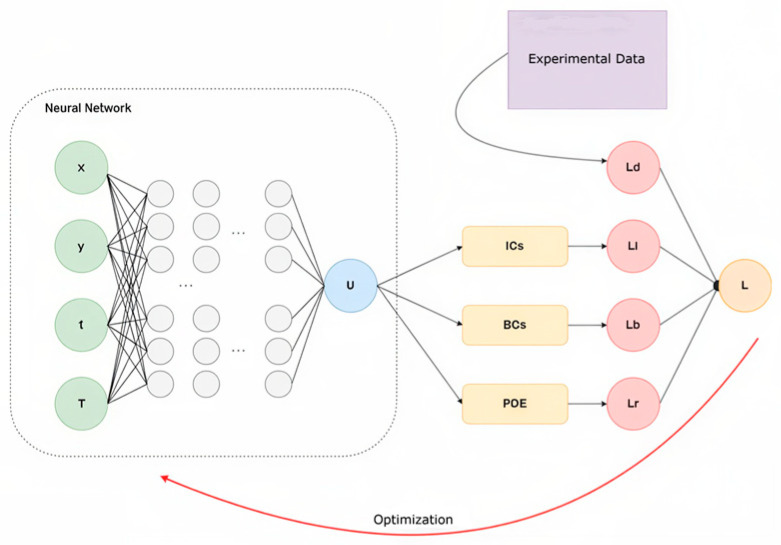
Workflow of the Physics-Informed Neural Network (PINN) architecture for temperature field modeling. Colors indicate functional components: green (inputs), blue (network output), purple (experimental data), beige (physical constraints), red (individual loss terms), and orange (total loss). Black arrows denote forward data flow, and the red curved arrow represents the optimization loop.

The physical constraints were defined using a model in which Q represents a moving Gaussian heat source, simulating the distribution of laser energy [34]. The neural network was provided with spatial coordinates (*x*, *y*), time t, and process parameters (laser power, scan speed, layer thickness) as inputs. It produced the predicted temperature field T_NN as output. As shown in Figure 4, the overall workflow of the proposed PINN framework is illustrated schematically.

The loss function combined data-driven and physics-informed components: [17]

(2) L=L_data+λ·L_physics

where L_data is the MSE between measured and predicted temperatures, and L_physics enforces the heat equation residual consistency:

(3) $$L_{physics}=\;{\left\|\;\rho \cdot cₚ\cdot \left(\frac{\partial T_{NN}}{\partial t}\right)-\;\nabla \cdot \left(k\nabla T_{NN}\right)-\;Q\;\right\|}_{2}^{2}$$

The weighting factor *λ* was determined empirically to balance the contributions of data-driven and physics-based loss terms during training. Thermophysical material properties (*ρ*, *c*ₚ, *k*) were treated as constants, with values adopted from representative literature for polyamide 12 (PA12).

Latent heat effects associated with polymer crystallization and temperature-dependent material properties were not explicitly modeled and are therefore considered a simplifying assumption of the present PINN formulation. This controlled simplification preserves the primary conductive heat-transfer behavior governing layer-wise thermal evolution, while avoiding additional model complexity that is not directly observable from the available infrared measurements. Modeling temperature-dependent material behavior was outside the scope of this study. This formulation maintains consistency with the dominant conductive heat-transfer mechanism and supports stable training behavior under limited data conditions. Training and evaluation used thermal data from 15 build cycles, collected via FLIR infrared recordings.

Each build cycle represented a distinct time segment of the polymer melting process. The model underwent 20,000 training iterations, with monitoring of RMSE, boundary conditions (BCs), initial conditions (ICs), and the partial differential equation (PDE). The established governing physics model is utilized, and the PINN component is incorporated into a multi-phase evaluation framework to enable physics-consistent thermal field prediction within the PBF-LB/P process.

Due to the computational cost of multi-physics training and inference, the PINN component is not intended for low-latency edge deployment. It is evaluated as an analytical complement to lightweight CNN-based detection.

### 2.7. Phase V: Experimental Evaluation of Lightweight Hybrid CNN Architectures

In the recent experimental stage, multiple lightweight convolutional neural network architectures were systematically designed, trained, and evaluated to determine the optimal trade-off among accuracy, generalization, and computational efficiency for real-time curling detection in PBF-LB/P systems. The primary objective of this phase was to quantify the trade-off between detection performance and inference latency under industrial edge-deployment constraints, rather than to maximize classification accuracy alone. All models were trained under identical fixed conditions (Table 2), employing 4-fold anti-leakage cross-validation, per-fold normalization, and class-balanced sampling with a weighted cross-entropy loss. Data partitioning strictly enforced non-overlapping temporal sequences and dedicated holdout isolation to ensure reproducibility and unbiased validation.

The selected architectures progressively reduce parameter count to systematically evaluate the trade-off between representational capacity and real-time inference latency. Four progressively compact convolutional neural network (CNN) variants were developed for this study: The variants comprise Pico CNN, which consists of two 2-dimensional convolutional (Conv2D) layers; Nonlight CNN, which includes three Conv2D layers; Nano CNN, which features three Conv2D layers with increased depth; and Microlite CNN, which incorporates three blocks that combine convolutional and depthwise operations, where depthwise convolutions process input channels independently. Each architecture was specifically designed to minimize parameter count while maintaining sufficient representational capacity. Performance was assessed using the area under the precision-recall curve (AUPRC), F1 score at threshold τ* (a statistical cutoff that balances precision and recall), precision, recall, and accuracy.

**Figure 5:**
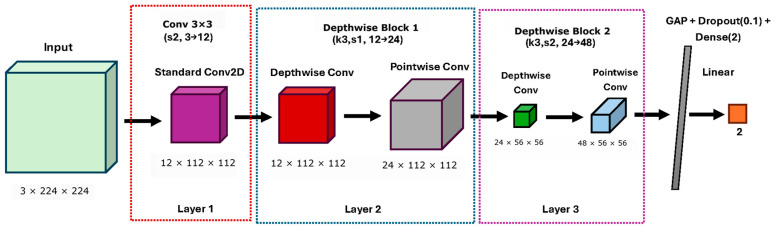
Microlite CNN (3 Layer Hybrid Conv + DW) Architecture. It combines standard and depthwise convolutions to reduce the parameter count (only 1.8 k) while maintaining high feature capacity, with ReLU activation applied after each convolutional layer. Colored blocks indicate architectural stages, and arrows represent forward feature flow.

These metrics were calculated across cross-validation folds and on an independent holdout set. Microlite CNN employs a hybrid architecture that integrates a standard 3 × 3 convolutional layer, followed by two depthwise-separable blocks [28] (DW Block), which are a combination of depthwise and pointwise convolutions with a kernel size of 3, strides of 1 and 2, and channel progression from 12 to 24 to 48. This is followed by a global average pooling (GAP) layer, dropout with a rate of 0.1, and a two-unit dense classifier. The architecture of the proposed Microlite CNN is illustrated in Figure 5, and its detailed layer configuration is summarized in Table 3.

## 3. Results

### 3.1. Phase I: Unsupervised Learning for Anomaly Detection

**Figure 6:**
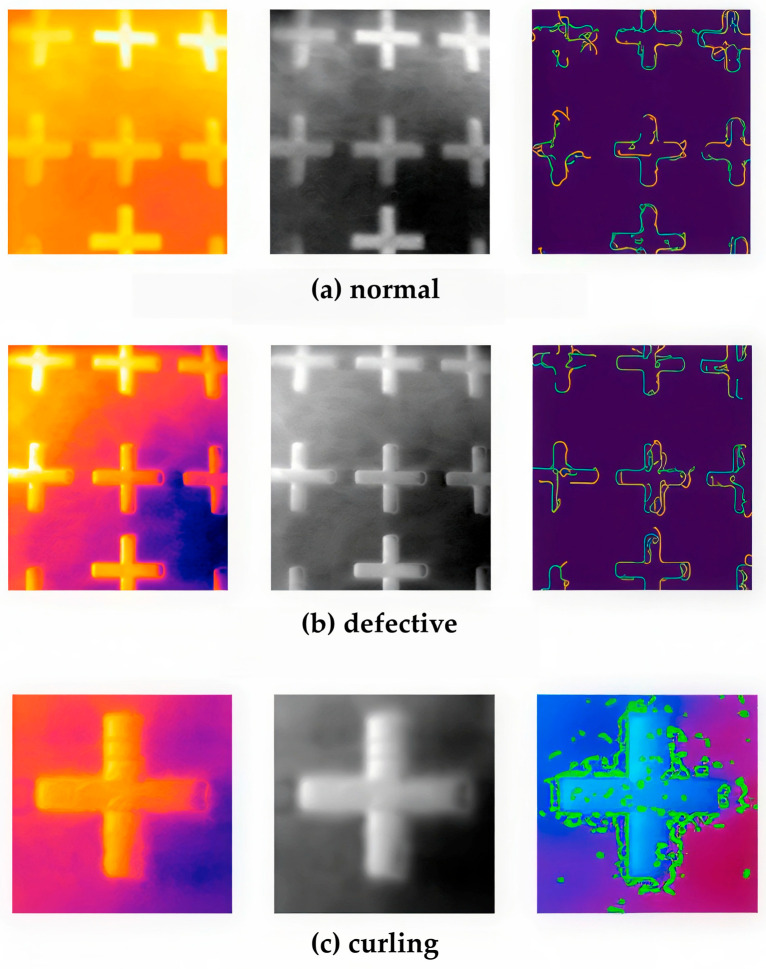
Representative thermal frames for (**a**) normal, (**b**) defective, and (**c**) curling samples. The left column shows the original thermal heatmaps, where warmer colors indicate higher temperature values. The middle column presents the grayscale intensity representation, and the right column illustrates the detected contour maps overlaid on the thermal field.

The autoencoder demonstrated the most consistent performance, combining stable training behavior with efficient inference. While GAN-based models achieved comparable detection accuracy (≈87%), they exhibited reduced stability and limited generalization across different build jobs. In contrast, classical unsupervised clustering approaches reported accuracies of up to 97%, underscoring the robustness of reconstruction-based and clustering-driven methods for real-time, unsupervised monitoring of thermal data in PBF-LB/P systems [37]. It should be noted that this high accuracy primarily reflects the dominance of nominal thermal patterns and does not necessarily imply sensitivity to subtle or early-stage defect manifestations. Representative examples of thermal responses under different conditions are shown in Figure 6.

However, although the method effectively identified contours on standard parts, distinguishing curling zones within irregular temperature distributions remained challenging because of thermal noise and variations in powder texture.

This method yielded basic structural information; however, its robustness was limited in comparison to the deep feature representations investigated in subsequent phases. This limitation highlights the restricted robustness of purely reconstruction-based methods under realistic thermal noise and motivates the transition toward learned deep feature representations in subsequent phases. The robust reconstruction consistency demonstrated by the autoencoder established a methodological basis for the subsequent supervised convolutional neural network (CNN) phase, which facilitated frame-level defect classification.

### 3.2. Phase II: Supervised CNN-Based Defect Classification

**Figure 7:**
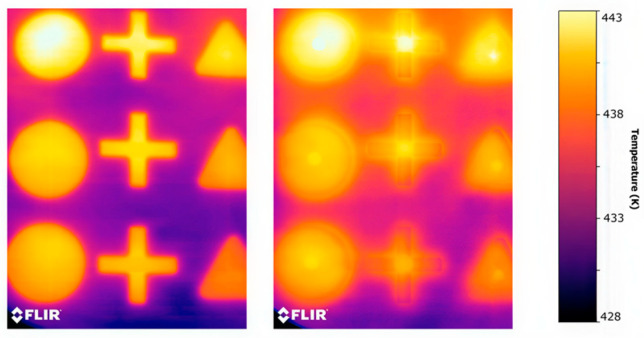
Examples of thermal images without defects (**left**) and with defects (**right**).

This section presents the experimental results of supervised CNN-based defect classification on thermal images acquired from the PBF-LB/P process. Representative defect-free and defective thermal samples are illustrated in Figure 7.

**Figure 8:**
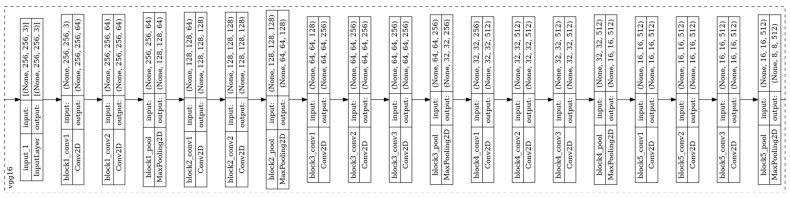
The VGG16 module used by the Trainer application [41]. The arrows represent the forward feature flow between network layers. This has been clarified in the caption.

Of all models evaluated, VGG-16 achieved the highest performance (accuracy = 99.09%, F1 score = 0.972) and demonstrated stable convergence. In contrast, ResNet50 and Xception exhibited significantly lower performance (approximately 16.6%) [24]. Since VGG-16 has significantly more parameters than ResNet50 and Xception, the difference might be explained by the “double descent” effect: larger deep-learning models may be easier to optimize than smaller ones and can perform well in the overparameterized regime because there are sufficiently many good local minima [38].The VGG16 architecture employed in this study is shown in Figure 8.

**Figure 9:**
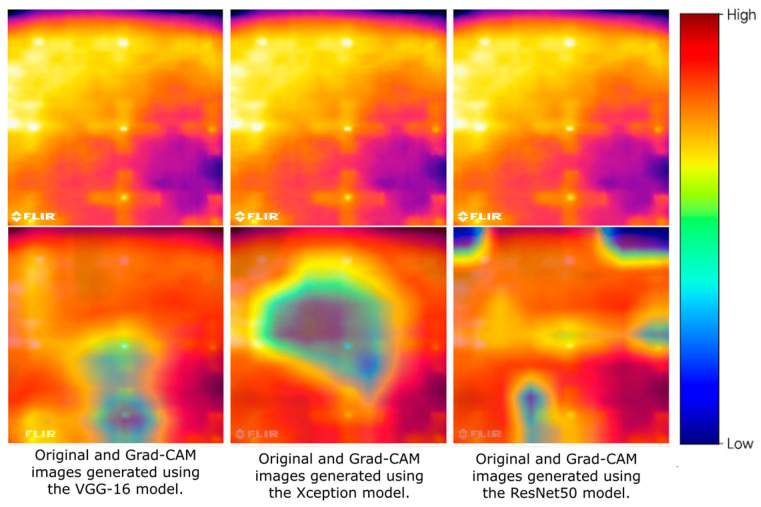
A visual comparison of Grad-CAM activations across the three convolutional neural network (CNN) architectures. This qualitative localization behavior is consistent with the quantitative performance trends reported in Table 4, reinforcing the link between spatial feature relevance and classification reliability.

Grad-CAM analysis confirmed that VGG-16 accurately focused on curling regions, supporting its selection as the most effective architecture among the evaluated models for defect classification in PBF-LB/P processes [20,39,40]. Grad-CAM activation maps for the evaluated models are shown in Figure 9.

The VGG-16 model exhibits strong localization in the curling region. Ref. [20], confirming that its learned features correspond to the actual temperature distortion observed on the powder bed. In contrast, the Xception and ResNet50 models exhibit dispersed or irrelevant activations, suggesting limited spatial generalization under the evaluated thermal imaging conditions.

These visual results are consistent with the quantitative metrics reported in Table 4, supporting the conclusion that VGG-16 achieves the optimal balance between accuracy and physical interpretability [20]. Reported accuracy values are presented with high numerical precision for comparative consistency; however, from a practical deployment perspective, variations within ±1% are considered operationally equivalent.

The prioritization of VGG-16 over deeper architectures was also theoretically motivated by the inherent signal characteristics of thermal IR imagery in PBF-LB/P. Unlike natural RGB datasets characterized by high-frequency edge gradients, thermal fields exhibit low-texture, diffuse heat distributions that necessitate stable, hierarchical feature extraction [3,42]. In such low-information-density domains, the extreme depth and residual skip-connections of architectures like ResNet50 can paradoxically induce feature vanishing or lead to overfitting on sensor noise [3], accounting for its suboptimal performance (16.58%). Conversely, simpler hierarchical structures provide a more stable representational bottleneck, a strategy effectively employed in recent complex recognition tasks [43]. This stability is particularly crucial in low-contrast environments where lightweight adaptive extraction and fusion strategies [44] have shown to outperform deep-layered models. Additional improvements might be achieved by incorporating dual-attention mechanisms or directional coordinate attention [43,44], which can effectively capture the macro-scale thermal gradients essential for defect identification without the vanishing gradient risks associated with excessive network depth. This specialized modeling approach aligns with the stringent manufacturability and structural constraints inherent in advanced additive manufacturing workflows [45]. Attention- or Transformer-based architectures were not included in the empirical benchmarking of this study. Such models typically entail substantially higher parameter counts and increased computational complexity, which may compromise low-latency inference and resource efficiency, key requirements for real-time process monitoring in industrial PBF-LB/P environments. Given that the proposed lightweight CNN architecture already achieves high predictive performance within stringent deployment constraints, the inclusion of significantly more complex architectures was not considered necessary for the targeted application scenario.

### 3.3. Phase III: Hybrid CNN-LSTM Architecture for Sequential Curling Detection

Although the single-frame CNN classifier demonstrated robust accuracy, it did not capture the temporal dynamics of curling progression. Consequently, a hybrid CNN-LSTM architecture was introduced to integrate sequential information across consecutive layers [32]. Following multiple iterations and dataset balancing, the hybrid model demonstrated strong generalization, achieving 97.64% accuracy, 100% precision, 47.08% recall, and an F1 score of 64.02% [41]. The relatively lower recall is primarily attributed to severe class imbalance and the conservative decision boundary adopted to minimize false positives in safety-critical industrial monitoring. These results indicate that the CNN-LSTM architecture effectively processes temporal curling patterns in video sequences and outperforms previous static CNNs in dynamic environments [32]. However, class imbalance and a limited number of defective samples contributed to reduced recall. Gradual improvement was observed after applying minority oversampling and removing irrelevant frames to reduce temporal noise and emphasize defect-relevant transitions, such as those depicting Rakel and laser actions. These findings demonstrate that integrating LSTM layers with CNNs enables effective spatio-temporal feature learning for in situ monitoring in PBF-LB/P [41]. Nevertheless, larger, more curated video datasets are needed to improve recall and further refine the model. This phase establishes a foundation for real-time, video-based defect prediction in additive manufacturing.

Although data-driven models demonstrated strong classification performance, their lack of physical interpretability remained a significant limitation. To overcome this, the final phase incorporated physics-based constraints using a Physics-Informed Neural Network (PINN) [17]. The results underscore the significance of temporal cues in curling detection and suggest the need for further investigation into lightweight temporal extensions. This motivates the integration of physics-informed constraints to complement data-driven temporal learning with physically consistent thermal predictions.

### 3.4. Phase IV: PINN Model Performance

Partial training results for the “Mid 1” cycle indicated consistent convergence across all loss components, with total loss decreasing from 1.859 × 10^1^ to 7.749 × 10^−2^ and RMSE reaching approximately 27 K. The final performance evaluation yielded the following results: a range of 1.859 × 10^1^ to 7.749 × 10^−2^ and an RMSE of approximately 27 K [46]. This error magnitude is consistent with reported infrared thermography uncertainties in polymer PBF-LB/P systems and remains acceptable for relative thermal trend monitoring. The detailed training metrics are presented in Table 5.

Final performance evaluation showed:

**Table 5:** The model’s training metrics, showing strong convergence (RMSE ↓ decreases from 1424 K to 27 K), stable learning after 2500 iterations, and high physical consistency with minor boundary errors.

Metric	Description	Observation
RMSE	Root Mean Squared Error	↓ from 1424 K → 27 K over training
BC/IC/PDE Loss	Boundary, Initial, and Equation residuals	Gradual convergence across 20 k iterations
Training Stability	RMSE trend and weighted loss	Stable and smooth after 2500 iterations
Physical Consistency	Compared with the measured data	High fidelity near the melt pool core; minor errors near boundaries

Note: (→) indicates change from initial to final value during training; (↓) denotes decrease.

The predicted temperature maps displayed smooth spatial distributions that closely corresponded to experimental thermal profiles. Most deviations were observed at locations with steep thermal gradients and at boundary regions, where convection, emissivity variations, and neglected latent heat effects are imperfectly represented. Overall, the PIML framework provided a physically consistent and data-efficient method for modeling the PBF-LB/P process. The framework effectively learned the thermal dynamics using limited labeled data and surpassed purely data-driven models in both robustness and interpretability. These findings underscore the feasibility of real-time, physics-constrained prediction for process monitoring and parameter optimization in additive manufacturing [18,46]. Several factors may limit the generalizability of this methodology. Thermal noise and variations in polymer powder emissivity can reduce radiometric accuracy, while minor misalignments in the camera’s optical axis may cause perspective distortions. Although anti-leakage splitting was applied for each build cycle, temporal drift in thermal patterns could still introduce bias. Additionally, even with double-checked expert labeling, manual annotations may introduce subjective uncertainty when distinguishing curling from delamination. Future work will address these limitations by expanding datasets, implementing automatic labeling pipelines, and applying domain adaptation techniques.

### 3.5. Phase V: Selection and Evaluation of Lightweight Hybrid Convolutional Neural Network Architectures

As shown in Table 6 and Figure 5, the baseline Pico CNN, although achieving high nominal accuracy, was unable to detect minority curling instances due to its limited feature depth (F1 = 0), where F1 is the harmonic mean of precision and recall. This behavior reflects extreme class imbalance rather than model instability. In comparison, both Nano CNN and Nonlight CNN demonstrated substantial improvements in recall (approximately 0.75) and overall robustness. Notably, the Microlite CNN architecture achieved an optimal balance between accuracy and computational efficiency. To contextualize the performance of lightweight architectures, more complex convolutional neural networks (CNNs), such as VGG-16, were evaluated in Phase II to establish an upper bound. This approach facilitates the interpretation of the performance-efficiency trade-off of Microlite CNN. Table 6 summarizes per-fold validation results and computational complexity indicators for the lightweight CNN architectures evaluated under the anti-leakage four-fold validation setting. For structural consistency, baseline models discussed in earlier phases were evaluated using a fixed validation split, whereas the four-fold protocol was applied in this phase to assess the robustness of the lightweight configurations. Performance metrics were computed using a fixed validation-based decision threshold, ensuring consistent benchmarking conditions across architectures. No post hoc threshold adjustment was applied during model comparison. Each architecture was independently trained and validated on balanced splits with non-overlapping build sequences. Strict data isolation was maintained, as verified in the Anti-Leakage Verification Report. The results indicate consistent generalization across folds, confirming the stability of the training process. Metrics such as F1@τ*, AUPRC, and τ* reflect the trade-off between precision and recall, while MACs, FLOPs, and latency indicate computational efficiency relevant to real-time PBF-LB/P monitoring.

As a result, this configuration reduces the parameter count to 1862 while maintaining high representational capacity (Figure 5). During the four-fold cross-validation phase, the Microlite CNN model consistently demonstrated robust, stable performance across all validation folds. The additional cross-validation analysis for Microlite-CNN confirmed stable performance trends across folds, indicating robustness of the lightweight architecture without significant variance fluctuations.

The average F1@τ* was 0.996, and the mean area under the precision–recall curve (AUPRC) was 0.988. These metrics demonstrate high discrimination performance between curling and non-curling frames. Reported metrics are presented with high numerical precision for consistency across folds; however, differences below ± 1% are considered practically equivalent in industrial deployment. The evaluation metrics on the independent test hold-out set are summarized in Table 7.

These results highlight Microlite CNN as the most computationally efficient architecture, achieving the lowest parameter count and fastest inference latency while maintaining comparable F1 and AUPRC scores. This level of performance confirms the model’s capacity to generalize within the validation partitions and maintain robustness across independent subsets. On the final anti-leakage holdout test set, the model maintained high performance, achieving an AUPRC of 0.932, F1@τ*_CV = 0.900, precision = 1.000, recall = 0.818, and accuracy = 0.998. These results validate the model’s transferability from the training phase to previously unseen build jobs, demonstrating high reliability and minimal false alarms in curling defect detection. High F1@τ* and AUPRC scores in both validation and holdout evaluations indicate a favorable balance between sensitivity and precision, underscoring Microlite CNN’s suitability for real-time, in situ quality monitoring in industrial PBF-LB/P processes.

The model demonstrated consistent performance across folds and rapid inference, with a CPU latency of 1.6 ms. This latency refers to pure inference time; acquisition and preprocessing overheads are hardware-dependent and discussed in the deployment considerations. Computational efficiency was assessed by quantifying the number of Multiply–Accumulate Operations (MACs) and Floating Point Operations (FLOPs). These metrics reliably estimate the model’s computational workload and potential throughput. Both MACs and FLOPs quantify the arithmetic workload for inference. Lower values correspond to greater computational efficiency and better suitability for real-time, resource-constrained deployment [47,48].

Among the architectures evaluated, Microlite CNN had the lowest complexity, requiring 11.29 million MACs and 22.58 million FLOPs. This result highlights it as the most lightweight model in the study.

On the final anti-leakage holdout set, performance increased under an operational deployment threshold, with an F1 score of 0.922 at τ_opt = 0.03, confirming the model’s robustness and reliability across previously unseen print jobs.

**Figure 10:**
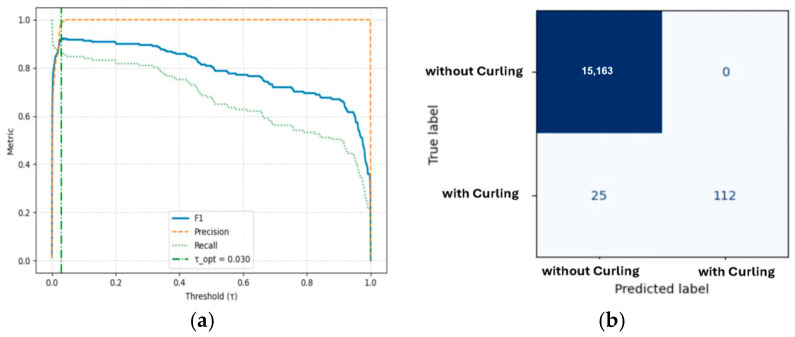
(**a**) Metric–threshold analysis illustrating the stability of F1, precision, and recall across validation thresholds. (**b**) Confusion matrix evaluated at the fixed validation-based decision threshold. Threshold sensitivity analysis was conducted for interpretative purposes only and did not influence model selection, training, or evaluation on independent data splits.

The confusion matrices (Figure 10, bottom row) indicated near-perfect true-negative classification and consistent defect recognition, with minimal false positives. These results provide strong evidence for the model’s suitability for industrial in situ deployment.

A previously reported customized convolutional neural network (CNN, a neural network particularly effective with image data) for thermal-based defect detection in PBF-LB/P achieved high accuracy with approximately 640,000 trainable parameters.

In comparison, Microlite CNN reduces the parameter count by more than 340 times while maintaining high interpretability, stability, and inference speed [23].

Furthermore, Microlite CNN is more than three times smaller and significantly faster than other CNN baselines with over 6000 parameters, while achieving comparable or significantly improved detection performance. Microlite CNN (a lightweight convolutional neural network) serves as a robust, efficient baseline for industrial defect monitoring, delivering high accuracy, efficiency, and reliability for real-time PBF-LB/P applications.

Performance is evaluated using two complementary thresholds: (i) a locked threshold τ*_CV, determined solely from cross-validation folds to ensure unbiased benchmarking, and (ii) an operational threshold τ_opt, derived from the holdout precision–recall curve for deployment-oriented tuning. Extremely low τ values observed in individual folds indicate severe class imbalance rather than model uncertainty. Therefore, AUPRC and fixed-threshold F1 are prioritized as the primary indicators of robustness.

## 4. Discussion

Deep learning offers considerable potential to enhance quality monitoring and predictive modeling in polymer powder bed fusion (PBF-LB/P). The study comprised five experimental phases, each using increasingly advanced neural models, ranging from unsupervised reconstruction methods to hybrid, lightweight architectures. Each approach demonstrated unique advantages, with some models providing higher accuracy and others offering improved interpretability or computational efficiency.

### 4.1. CNN-Based Defect Classification

Among the supervised models evaluated, the fine-tuned VGG-16 network demonstrated the highest stability and accuracy, achieving 99.09% accuracy and an F1 score of 0.972 in detecting curling defects in thermal images. In contrast, architectures such as ResNet-50 and Xception failed to generalize effectively under conditions of severe class imbalance. This can be explained by the “double descent” effect: larger deep-learning models may be easier to optimize than smaller ones and can perform well in the overparameterized regime because there are sufficiently many good local minima [38]. These results suggest that, for small industrial datasets, simpler models combined with effective data conditioning offer greater advantages than increased architectural complexity. Grad-CAM visualizations further confirmed that VGG-16 accurately localized curling regions, supporting its suitability for precise, rapid quality control. Collectively, these findings highlight the importance of supplementing overall accuracy with risk-sensitive metrics, including recall and false-negative rate, particularly in safety-critical applications. The observed underperformance of ResNet-50 and Xception is consistent with prior studies on the PBF-LB/P thermal dataset, where these architectures experienced generalization collapse despite strong results on natural image benchmarks. This phenomenon is attributed to a domain mismatch between ImageNet-pretrained representations and low-texture infrared imagery, exacerbated by severe class imbalance and near-duplicate frame distributions.

### 4.2. Temporal Modeling with CNN-LSTM

The hybrid CNN-LSTM model effectively captures the temporal evolution of curling across consecutive layers, demonstrating notable success. The model achieved 97.64% accuracy and 100% precision; however, recall remained moderate at 47.08%, indicating a limited ability to detect all relevant anomalies. This behavior characterizes the model as a conservative detector, highly reliable for severe anomalies but less responsive to early-stage irregularities. Such behavior is acceptable in industrial monitoring contexts where false positives incur high operational cost, and where confirmation of severe defects is prioritized over early but uncertain detection. Although sequence modeling provides valuable temporal context, its performance is constrained by class imbalance and the limited number of defect examples, which may restrict generalizability and early-stage anomaly detection. Time-aware models should complement, rather than replace, single-frame CNN classifiers in process monitoring applications. Although random undersampling (RUS) and oversampling (ROS) were applied, the temporal imbalance associated with progression limited recall. Consequently, in its current form, the CNN–LSTM architecture primarily serves as a reliable tool for confirming severe defects, rather than as an early warning system for less evident issues. Future research will explore the use of CNN–LSTM models for defect prediction, along with synthetic temporal augmentation and domain adaptation to increase sensitivity to early-stage defects.

### 4.3. Unsupervised and Generative Approaches

Unsupervised anomaly detection reduced dependence on labeled data.

Subsequently, a semi-supervised K-Means plus classifier framework achieved 99.7% accuracy, surpassing standalone autoencoders (AEs) and Generative Adversarial Networks (GANs), which attained approximately 87% accuracy due to instability and mode collapse. Although reconstruction-based methods enable real-time inference, their generalization remains limited. Additionally, combining unsupervised learning methods such as K-Means clustering and autoencoders with supervised learning can improve defect detection performance in use cases with a limited amount of labeled data.

### 4.4. Physics-Informed Neural Networks (PINNs)

The Physics-Informed Neural Network (PINN) incorporates heat-transfer equations directly within its loss function. Based on this approach, the model achieved a root-mean-square error (RMSE) of approximately 27 K across multiple build cycles and reduced computational cost by approximately 70% compared to finite-element analysis. Furthermore, this dual capability for forward prediction and inverse parameter estimation highlights the potential of PINNs in process control and predictive maintenance.

Additionally, the model generated smooth and physically consistent temperature fields, supporting its feasibility for real-time, physics-constrained inference. The PINN component serves as a reduced-order, physics-consistent surrogate rather than a high-fidelity melt-pool solver. This design choice intentionally favors computational efficiency and physical consistency over fine-scale accuracy. Its primary objective is to capture the relative spatio-temporal evolution of thermal fields at the layer scale, enabling physically plausible trend-level monitoring suitable for real-time deployment. Future work will address incorporating temperature-dependent material properties, latent heat of crystallization, and higher-fidelity boundary modeling.

A semi-quantitative comparison reveals that defect-labeled (curling) frames exhibit significantly higher spatial thermal variance than non-defective frames (*p* < 0.01), indicating amplified temperature heterogeneity during layer consolidation. Prior synchronized thermographic–profilometric investigations conducted on the same experimental platform (our previous work [49]) reported curling magnitudes in the range of approximately 23–80 μm, spatially co-localized with thermal inhomogeneities. While the present study does not directly measure out-of-plane deformation, the statistically elevated thermal fluctuation metrics align with these previously quantified deformation ranges, thereby supporting a physically plausible linkage between thermal instability and defect manifestation without implying a direct linear correspondence.

### 4.5. Lightweight Hybrid CNN Architectures

During the final phase, four compact convolutional neural network (CNN) architectures were systematically evaluated using identical four-fold anti-leakage cross-validation. Among the evaluated architectures, the proposed Microlite CNN demonstrated the most favorable balance of performance metrics, achieving an area under the precision-recall curve (AUPRC) of 0.988 and an F1 score of 0.996 on the validation set, as well as an F1 score of 0.90 and an AUPRC of 0.932 on the holdout set. For clarity, all comparative claims are based on the locked validation threshold (τ*_CV). Any additional operating thresholds are reported solely for deployment interpretation and were not used for model comparison.

Despite utilizing only 1860 parameters, the model maintained strong generalization and robustness. The model required 11.3 million Multiply–Accumulate Operations (MACs) and 22.6 million floating-point operations (FLOPs), resulting in the lowest computational load and the fastest central processing unit (CPU) latency of 1.6 milliseconds. As a result, this configuration was over 300× lighter than previous thermal CNNs, confirming its suitability for real-time, resource-constrained industrial deployment.

### 4.6. Synthesis and Practical Implications

Each methodological phase systematically built a progressively stronger hybrid framework. Specifically, CNNs ensured high spatial accuracy, while LSTMs captured temporal dynamics. In parallel, unsupervised methods enhanced data efficiency, and PINNs introduced physical consistency. Bringing these elements together, the Microlite CNN unified these advances, achieving highly reliable defect discrimination with minimal computational cost. Taken together, these findings demonstrate that lightweight, physics-guided hybrid deep-learning models can deliver scalable, efficient, and interpretable AI solutions for PBF-LB/P systems. A detailed quantitative comparison of model performance is provided in Table 8.

To further contextualize the proposed deep-learning–based framework, a comparison with established sensor-based in situ monitoring approaches is warranted. In particular, laser profilometry combined with thermal imaging has been demonstrated as an effective method for analyzing curling defects in PBF-LB/P processes [49]. While such approaches provide high geometric accuracy through direct surface deformation measurements correlated with thermal signatures, they require additional hardware integration, precise calibration, and increased system complexity, which may limit scalability and industrial adoption. The comparative analysis further highlighted the inherent training instabilities of GAN-based anomaly detection, such as mode collapse and vanishing gradients [50,51]. This justified the transition toward the more stable, hierarchical feature learning observed in our optimized Microlite–CNN architecture. This architectural shift ensures consistent convergence even in the presence of the stochastic thermal fluctuations typical of polymer PBF-LB/P processes.

In contrast, the framework presented in this study achieves competitive defect detection performance using camera-based sensing combined with lightweight convolutional neural networks, without reliance on external profilometry hardware. The proposed Microlite CNN demonstrates that comparable diagnostic capability can be obtained through data-driven learning while significantly reducing system complexity, computational overhead, and deployment costs. This comparison highlights the trade-off between hardware-intensive measurement precision and software-driven scalability, where the latter offers a more flexible and cost-efficient pathway toward real-time, industrial-grade quality monitoring in PBF-LB/P systems. A consolidated summary of the experimental results across Phases I–V is presented in Table 9.

Direct end-to-end runtime measurements are not reported for all evaluated models. Instead, model complexity indicators, particularly the number of trainable parameters, serve as proxies for computational efficiency because parameter count directly influences inference runtime. Although parameter count does not account for all hardware-specific nuances, it serves as a consistent and widely recognized proxy for comparative computational efficiency across diverse architectures. Notwithstanding the high predictive fidelity of the proposed framework, it is acknowledged that industrially critical variables, principally atmospheric humidity and oxidative powder degradation, represent external sources of variability that were held constant within the present experimental scope. Integrating multi-modal environmental sensing in future iterations could further enhance process stability by compensating for stochastic drifts within the sintering window. Furthermore, the achieved millisecond-scale inference establishes a robust computational foundation for potential closed-loop intervention, enabling strategies such as localized laser power modulation or autonomous process suspension to mitigate the risk of catastrophic part failure

## 5. Conclusions and Outlook

This study presents a multi-phase framework for defect detection and thermal behavior modeling in PBF-LB/P, integrating data-driven and physics-informed deep-learning approaches. The investigation comprised five experimental stages and yielded three principal outcomes: (i) the creation of a reproducible in situ dataset accompanied by a rigorous anti-leakage protocol, (ii) the development of lightweight hybrid convolutional neural network (CNN) models for real-time curling detection, and (iii) the application of physics-informed learning for thermally consistent behavior prediction.

The proposed Microlite CNN attained competitive detection performance while utilizing over 300× fewer parameters than comparable methods. This demonstrates that energy-efficient and interpretable artificial intelligence can satisfy rigorous industrial timing requirements. Collectively, the results establish a robust and lightweight baseline for real-time curling detection in PBF-LB/P processes and provide evidence of strong generalization across independent build jobs within the evaluated dataset. These findings emphasize the feasibility of scalable, reliable, and sustainable deep-learning-driven quality monitoring in industrial polymer additive manufacturing. Future work will focus onthe detection of other process defects, defect prediction, and real-time process parameter optimization.

## Figures and Tables

**Table 1:** Technical specifications of the EOS FORMIGA P 110 system (EOS, Krailling, Germany).

Item	Technical Spec
Laser type	CO_2_
Laser power	30 W
Laser wavelength	10.6 µm
Scanning speed	5 m/s
Layer thickness	50–200 µm
Powder type	PA2200 nylon

**Table 2:** Fixed Parameters on Training Models.

Parameter	Description
K-Fold	4
Split Protocol	Fixed manifest (anti-leakage per build)
Normalization	Per-fold mean/std
Input Resolution	224 × 224
Channels	3 (RGB)
Transform	Resize → To Tensor → Normalize (mean, std)
Augmentation (Train only)	H Flip (0.5) + Rotation (±10°) + Color Jitter (0.2, 0.2)
Class Balance	Weighted loss
Batch Size	32
Optimizer	Adam W [35,36] (lr = 5 × 10^−4^, wd = 1 × 10^−4^)
LR Scheduler	Cosine Annealing LR (Tₘₐₓ = 30)
Epochs	30 (Early stop: patience = 7)
Threshold Selection	τ* (F1-optimal on validation)
Metrics	F1@τ*, AUPRC, Accuracy
Device	GPU for training, CPU for latency evaluation
Loss Function	Weighted Cross-Entropy [30]

Note: τ* denotes the validation-optimized threshold and is used as a unified symbol throughout the manuscript.

**Table 3:** Detailed architecture of the Microlite CNN. The symbol ‘→’ denotes the mapping from input to output channels.

Block	Layer Type	Kernel	Stride	Padding	In → Out Channels	Output Size	Parameters
Input	RGB Image	–	–	–	3	224 × 224	–
B1	Conv2D	3 × 3	2	1	3 → 12	112 × 112	336
B1	Depthwise Conv (DWConv)	3 × 3	1	1	12 → 12	112 × 112	108
B1	Pointwise Conv (PWConv)	1 × 1	1	0	12 → 24	112 × 112	288
B2	Depthwise Conv (DWConv)	3 × 3	2	1	24 → 24	56 × 56	216
B2	Pointwise Conv (PWConv)	1 × 1	1	0	24 → 48	56 × 56	768
Head	Global Average Pooling (GAP)	–	–	–	48	1 × 1	0
Head	Fully Connected (FC)	–	–	–	48 → 2	1 × 1	66

Note: The symbol ‘→’ denotes the mapping from input to output channels.

**Table 4:** Evaluation of Different Pretraining CNN Models. Low performance of deeper architectures reflects transfer limitations under severe class imbalance and low-texture thermal imaging conditions.

Model	Pretraining	Parameters (M)	Accuracy (%)	F1-Score	Key Observations
VGG-16	ImageNet	138	99.09	0.972	High generalization; stable training; strong curling detection
ResNet50	ImageNet	25.6	16.58	0.165	Failed to converge; vanishing gradients.
Xception	ImageNet	22.9	16.58	0.165	Poor transfer; depthwise filters are ineffective

**Table 6:** Comparison of cross-fold performance and computational complexity for lightweight convolutional neural network (CNN) architectures using anti-leakage 4-fold validation. Bolded values represent the average results across folds for each architecture. CPU latency was measured on an Intel Core i7-6700K processor using single-thread inference.

Model	Channel	Params	MACs	FLOPs	CPU Lat. (ms)	Val F1@τ*	Val AUPRC	Val τ*
Pico CNN (2 Conv2D Layers)	3 → 12 → 24	2966	12,192,816	24,385,632	5.8			
Fold 1						0.355	0.438	0.35
Fold 2						0.610	0.412	0.50
Fold 3						0.703	0.554	0.55
Fold 4						0.563	0.453	0.17
Nano Light CNN (3 Conv2D Layers)	6 → 12 → 24	3452	6,096,432	12,192,864	2.1			
Fold 1						0.996	0.993	0.01
Fold 2						0.894	0.904	0.16
Fold 3						0.976	0.950	0.48
Fold 4						0.996	0.985	0.02
Nano CNN (3 Conv2D Layers)	8 → 16 → 32	6042	9,934,912	19,869,824	2.2			
Fold 1						0.996	0.986	0.91
Fold 2						0.889	0.833	0.03
Fold 3						0.976	0.946	0.03
Fold 4						0.996	0.985	0.01
Micro Lite CNN (Hybrid Conv + DW)	12 → 24 → 48	1862	11,289,696	22,579,392	1.6			
Fold 1						0.996	0.988	0.15
Fold 2						0.889	0.882	0.20
Fold 3						0.976	0.949	0.68
Fold 4						0.996	0.985	0.01

Note: The arrow (→) indicates the progression of feature channel dimensions across successive convolutional layers.

**Table 7:** Evaluation of Model Metrics and Results on Test Hold Out.

Model	Channel	Params	τ*_CV	AUPRC	F1@τ*_CV	Precision	Recall	Accuracy
Pico CNN (2 Conv2D Layer)	3 → 12 → 24	2966	0.500	0.140	0.000	0.000	0.000	0.991
Nonlight CNN (3 Conv2D Layer)	6 → 12 → 24	3452	0.161	0.920	0.858	1.000	0.752	0.998
Nano CNN (3 Conv2D Layer)	8 → 16 → 32	6042	0.247	0.900	0.834	1.000	0.750	0.997
Microlite CNN (Hybrid Conv + DW)	12 → 24 → 48	1862	0.251	0.932	0.900	1.000	0.818	0.998

Note: The arrow (→) denotes the progression of feature channel dimensions across successive convolutional layers.

**Table 8:** Detailed quantitative evaluation of model metrics.

Model	Accuracy (%)	Recall (%)	F1 Score	RMSE (K)	Params
autoencoder/GAN/K-Means	97.0 (K-Means + Clf)	90.5	0.91	–	~50 k (est.)
VGG-16 CNN	99.09	95.9	0.972	–	138 M
CNN-LSTM Hybrid	97.64	47.1	0.64	–	25 M
Physics-Informed NN (PINN)	–	–	–	27 ↓	8 M
Microlite CNN (Hybrid Conv + DW)	99.8 (val)/99.7 (test)	81.8	0.900 (test)/0.996 (val)	–	1.86 k
Custom CNN (low-cost RGB) [23]	>99 (Acc, Prec, Rec)	99.1	0.992	–	640 k

Note: (↓) indicates a decrease in RMSE relative to the initial training error.

**Table 9:** Experimental Results Across Phases I–V.

Phase	Method	Task	Dataset	Key Metrics	Main Strength	Limitation
I	autoencoder/GAN/K-Means	Unsupervised anomaly detection	Thermal images	Acc ≈ 97% (K-Means + Classifier)	Works with minimal labels	Dataset-specific; GAN instability
II	VGG-16 CNN	Frame-based defect classification	Thermal frames	Acc = 99.09%; F1 = 0.972	High accuracy; robust optimization in overparametrized regions	Needs labeled data; sensitive to imbalance
III	CNN-LSTM Hybrid	Sequence-based defect detection	IR video	Acc = 97.64%; Prec = 100%; Rec = 47.1%	Captures temporal correlations	Misses subtle defects
IV	PINN (PIML)	Thermal field prediction	IR + Simulated data	RMSE ≈ 27 K; 70% faster than FEM	Physics-constrained; data-efficient	Parameter determination, computational effort
V	Microlite CNN (Hybrid Conv + DW)	Real-time curling detection	RGB 4-fold cross-val + holdout	F1 = 0.996 (val), 0.90 (test); Latency = 1.6 ms	Ultra-lightweight; robust generalization	Limited to RGB/thermal inputs, other defects under different optical conditions might be difficult to detect

## Data Availability

The raw data supporting the conclusions of this article will be made available by the authors on reasonable request.

## Abbreviations

The following abbreviations are used in this manuscript:

AM	Additive Manufacturing
DL	Deep Learning
CNN	Convolutional Neural Network
RNN	Recurrent Neural Network
LSTM	Long Short-Term Memory
GAN	Generative Adversarial Network
PINN	Physics-Informed Neural Network
PBF-LB/P	Powder Bed Fusion—Laser Beam of Polymers
PBF-LB/M	Powder Bed Fusion—Laser Beam of Metals
AE	Autoencoder
PDE	Partial Differential Equation
PIML	Physics-Informed Machine Learning
IR	Infrared
PA12	Polyamide 12
PP	Polypropylene
TPU	Thermoplastic Polyurethane
RUS	Random Undersampling
ROS	Random Oversampling
Acc	Accuracy
F1	F1 Score
RMSE	Root Mean Square Error
AUPRC	Area Under the Precision–Recall Curve
XAI	Explainable Artificial Intelligence
Grad-CAM	Gradient-weighted Class Activation Mapping
GAP	Global Average Pooling
DWConv	Depthwise Convolution
PWConv	Pointwise Convolution
CPU	Central Processing Unit
GPU	Graphics Processing Unit
FEM	Finite Element Method
FLOPs	Floating-Point Operations
MACs	Multiply–Accumulate Operations
τ*	Validation-Optimal Threshold
